# *Piper sarmentosum* Roxb. Attenuates Vascular Endothelial Dysfunction in Nicotine-Induced Rats

**DOI:** 10.3389/fphar.2021.667102

**Published:** 2021-06-14

**Authors:** Muhd Fakh Rur Razi Md. Salleh, Amilia Aminuddin, Adila A. Hamid, Norizam Salamt, Fadhlullah Zuhair Japar Sidik, Azizah Ugusman

**Affiliations:** ^1^ Department of Physiology, Faculty of Medicine, Universiti Kebangsaan Malaysia, Cheras, Malaysia; ^2^ Department of Pharmacology, Faculty of Medicine, Universiti Kebangsaan Malaysia, Cheras, Malaysia

**Keywords:** nicotine, nitric oxide, oxidative stress, vascular remodeling, vascular endothelial dysfunction, *Piper sarmentosum* Roxb.

## Abstract

Exposure to cigarette smoke is an important risk factor for cardiovascular diseases. Nicotine is an addictive compound in cigarette smoke that triggers oxidative stress, which leads to vascular dysfunction. *Piper sarmentosum* Roxb. is a herb with antioxidant and vascular protective effects. This study evaluated the potential protective effect of the aqueous extract of *P. sarmentosum* leaf (AEPS) on vascular dysfunction in rats induced with prolonged nicotine administration. A total of 22 male Sprague-Dawley rats were divided into control (normal saline, oral gavage [p.o.]), nicotine (0.8 mg/kg/day nicotine, intraperitoneally [i.p.]), and nicotine + AEPS groups (250 mg/kg/day AEPS, p.o. + 0.8 mg/kg/day nicotine, i.p.). Treatment was given for 21 days. Thoracic aortae were harvested from the rats for the measurement of vasorelaxation, vascular nitric oxide (NO) level, and antioxidant level and the assessment of vascular remodeling. Rats treated with AEPS had improved vasorelaxation to endothelium-dependent vasodilator, acetylcholine (ACh), compared with the nicotine-induced rats (*p* < 0.05). The presence of endothelium increased the maximum relaxation of aortic rings in response to ACh. Compared with the nicotine group, AEPS enhanced vascular NO level (*p* < 0.001) and increased antioxidant levels as measured by superoxide dismutase activity (*p* < 0.05), catalase activity (*p* < 0.01), and reduced glutathione level (*p* < 0.05). No remarkable changes in aortic histomorphometry were detected. In conclusion, *P. sarmentosum* attenuates vascular endothelial dysfunction in nicotine-induced rats by improving vasorelaxation and enhancing vascular NO and antioxidant levels.

## Introduction

Tobacco smoking contributes to eight million of annual deaths worldwide. More than seven million deaths are attributed to the direct effects of smoking, and more than 1.2 million premature deaths of adults and children due to exposure to secondhand smoke have been recorded annually (World Health Organization, 2019). Nicotine is the main ingredient in tobacco smoke that causes addiction (Hecht, 2003). In addition, tobacco smoking and exposure to nicotine increase the risk of cardiovascular diseases (CVD), such as atherosclerosis, ischemic heart disease, hypertension, and stroke (Son and Lee, 2020)*.*

The effect of nicotine on the cardiovascular system is mediated by its binding to endogenous nicotinic acetylcholine receptors (nAChRs). NAChRs are expressed by vascular endothelial cells (EC) and vascular smooth muscle cells (VSMC); thus, ECs and VSMCs are the direct targets of nicotine-induced vascular dysfunction (Brüggmann et al., 2003; Moccia et al., 2004). The presentations of nicotine-induced vascular dysfunction include changes in vasoreactivity and vascular remodeling (Whitehead et al., 2021).

Nicotine impacts the survival, proliferation, migration, and matrix production of ECs and VSMCs and leads to vascular remodeling. Acute exposure to nicotine promotes the angiogenic response of the endothelium, whereas chronic exposure blunts pro-angiogenic response. Acute and chronic nicotine exposures lead to fibroblast proliferation, extracellular matrix deposition, VSMC proliferation and migration, and neointima formation (Cucina et al., 2000; Ng et al., 2007; Rodella et al., 2012). In addition, chronic nicotine infusion induces matrix metalloproteinase (MMP)-2 and MMP-9 expression and activity. MMPs cause elastin degradation in the aortic wall and irreversible aortic stiffening (Wagenhauser et al., 2018).

Concerning vascular reactivity, nicotine impairs vasorelaxation through endothelium-dependent and -independent mechanisms. The endothelium secretes various vasoactive substances that move to the underlying VSMC to induce vasoconstriction or vasorelaxation (Wright et al., 2004). Nicotine stimulates the release of the vasoconstrictor, endothelin-1, and inhibits the synthesis of the vasorelaxants, nitric oxide (NO) and prostacyclin, from the endothelium (Su and Wang, 1991; Toda and Toda, 2010; Durand and Gutterman, 2013). As for the endothelium-independent mechanisms, nicotine promotes vasoconstriction by activating voltage-gated potassium channels and enhancing VSMC response to norepinephrine (Mayhan and Sharpe, 2002; Olfert et al., 2018).

Oxidative stress is the underlying pathogenesis of nicotine-induced impaired vasorelaxation (Carnevale et al., 2016). Nicotine stimulates excessive reactive oxygen species (ROS) production and lowers antioxidant levels to cause oxidative stress (Tonnessen et al., 2000). Multiple studies suggested that excessive ROS contributes to the nicotine-induced impairment of NO-mediated, endothelium-dependent vasorelaxation (Jiang et al., 2006; Toda and Toda, 2010). Cells produce a system of endogenous antioxidants to neutralize ROS. Among these antioxidants are superoxide dismutase (SOD), catalase (CAT), and reduced glutathione (GSH) (Aguilar et al., 2016). Nicotine exposure reduces aortic SOD and GSH levels, and this finding is associated with the impairment of the endothelium-dependent vasorelaxation of the aorta (Zainalabidin et al., 2014).

*Piper sarmentosum* Roxb. is a herbaceous plant that is widely used in Chinese traditional medicine to treat fever, cough, and pleurisy. Pharmacologically, the herb has various vascular protective effects (Peungvicha et al., 1998; Mohd Zainudin et al., 2013). The aqueous extract of *P. sarmentosum* leaf (AEPS) has high antioxidant activity (Ismail et al., 2018) and can stimulate NO production in oxidative stress-induced endothelial cells (Ugusman et al., 2010). AEPS also reduces the formation of atherosclerotic lesions in hypercholesterolemic rabbits (Amran et al., 2010).

Even though AEPS has vascular protective effects in various experimental models, the potential of AEPS in attenuating nicotine-induced vascular dysfunction, including impaired vasorelaxation and vascular remodeling, has not been studied. Nicotine in tobacco smoke causes direct harm to smokers and, unfortunately, has harmful effects on passive smokers or people who are inadvertently exposed to environmental tobacco smoke (World Health Organization, 2019). We hypothesized that AEPS can protect against the vascular dysfunction caused by nicotine. Therefore, this study determined the effect of AEPS on vasorelaxation, vascular NO level, and antioxidant levels, as well as vascular remodeling, in nicotine-induced rats. Findings from this study will support the potential use of AEPS as a supplement to prevent nicotine-induced vascular dysfunction in people who are inadvertently exposed to tobacco smoke.

## Materials and Methods

### Preparation and Analysis of Aqueous extract of *Piper sarmentosum* Roxb. leaf

*P. sarmentosum* leaves were supplied by Herbagus Sdn. Bhd., Penang, Malaysia and identified by a plant taxonomist in the Herbarium of Universiti Kebangsaan Malaysia (UKM; specimen voucher number: UKMB40240). AEPS was prepared according to a previous method (Ugusman et al., 2011). Fresh *P. sarmentosum* leaves were sun-dried and ground into powder. The powder was mixed with distilled water in a ratio of 1:10 (w/v) and heated at 80°C for 3 h. The extract was filtered and concentrated repeatedly and then freeze-dried and kept at 4°C. Liquid chromatography–mass spectrometry (LCMS)–Orbitrap full-scan analysis was conducted to identify the compounds in AEPS, and the results have been published previously (Sundar et al., 2019).

### Animals and Study Design

The study was approved by the Animal Ethics Committee of UKM (approval code: PP/FISIO/2018/AZIZAH/26-SEPT./957-SEPT.-2018-SEPT.-2019). Twenty-two male Sprague-Dawley rats (250–300 g) were obtained from the Animal Resource Unit of UKM. Each rat was kept in a cage and maintained under standard conditions of a 12 h light and 12 h dark cycle. The rats were fed on a standard rat chow diet with water ad libitum. The rats were randomly divided into three groups (*n* = 6–8 per group): the control group was given normal saline; the nicotine group was given 0.8 mg/kg/day nicotine (Tokyo Chemical Industry, Japan) intraperitoneally (i.p.); and the AEPS group was fed with 250 mg/kg/day AEPS by oral gavage (p.o.) 30 min before treatment with 0.8 mg/kg/day nicotine (i.p.). Treatment was continued daily for 21 days. The dosage and duration of nicotine treatment mimic the exposure of a chronic light smoker and has been proven to cause vascular dysfunction in a previous study (Moon et al., 2014). The dose of AEPS was chosen based on the optimal dose that improved vasorelaxation in nicotine-induced rats (Supplementary Figure S1, Supplementary Table S1). On day 22, the rats were terminally anaesthetized with intravenous injection of ketamine and xylazine cocktail (0.2 ml/kg BW). The thoracic aortae were then harvested and cleaned from the surrounding fat and connective tissues. Parts of the fresh aortic tissues were immediately used for wire myography. The remaining aortic tissues were used for vascular NO, antioxidant, and histological analyses.

### Measurement of Mean Systolic Blood Pressure

MSBP was measured in conscious rats on day 21 by using the CODA II™ Non-Invasive Blood Pressure System (Kent Scientific Corporation, United States). MSBP values were then used for aortic morphometry analysis.

### Wire Myography

The thoracic aortae were cut into 2 mm rings. Some rings were endothelium-denuded, whereas others had intact endothelium. The endothelium was removed by gently rubbing the interior of the vessel around a wire, and removal was confirmed by the lack of a vasodilator response to 10^–6^ M acetylcholine (ACh; Tokyo Chemical Industry, Japan). The aortic rings were mounted on two stainless steel pins in a four-channel wire myograph (Danish Myo Technology, United States). The vessels were bathed in physiological Krebs solution with the following composition: 118 mM NaCl, 4.7 mM KCl, 11 mM glucose, 1.2 mM MgSO_4_, 25 mM NaHCO_3_, 1.03 mM KH_2_PO_4_, and 2.5 mM CaCl_2_. Then, the vessels were gassed continuously with 95% O_2_ and 5% CO_2_ at 37°C. The aortic rings were set to an optimum tension of 1 g and allowed to equilibrate for at least 30 min before use (Almabrouk et al., 2018). After the calibration, the viability of the aortic rings was tested by adding 40 mM KCl. The vessels were then contracted with 10^–6^ M phenylephrine (Sigma, United States) before starting experiments. The cumulative concentration–relaxation curves to the endothelium-dependent vasodilator, ACh (10^–9^–10^–6^ M), and the endothelium-independent vasodilator, sodium nitroprusside (SNP; 10^–9^–10^–6^ M; Sigma, United States), added at 3 min intervals were constructed. Power-Lab Data Acquisition System (ADInstruments, Australia) was used to measure and record the changes in vessel tension. Vasorelaxation data were expressed as the percentage loss of phenylephrine-induced contraction.

### Preparation of Tissue Lysates

Aortic tissue lysates were prepared based on previous methods (Zainalabidin et al., 2014). The tissues were weighed and crushed into powder form using a mortar and pestle in liquid nitrogen. Then, phosphate-buffered saline (PBS; 0.1 M, pH 7.4) was added to the tissue powder in a ratio of 1:9 (w/v) and centrifuged for 10 min at 4°C. The protein concentration in the aortic tissue lysates was measured using Bradford assay (Bradford, 1976).

### Measurement of Vascular Nitric Oxide Level

The concentration of NO in the aortic tissue lysates was measured indirectly using Nitrite/Nitrate Colorimetric Assay Kit (Sigma, United States) according to the manufacturer’s instructions. The whole aorta was used for this assay without specifically isolating the endothelial layer. The principle of this assay is based on the measurement of total nitrite in the samples. Nitrate reductase was used to reduce nitrate to nitrite. Total nitrite was measured at 540 nm after the addition of Griess reagent.

### Measurement of Vascular Antioxidant Levels

SOD activity in the aortic tissue lysates was measured as described previously (Beyer and Fridovich, 1987). Briefly, the tissue lysates were mixed with SOD substrate containing PBS–EDTA, riboflavin, L-methionine, Triton-X, and nitro blue tetrazolium (NBT). SOD activity in the samples was determined on the basis of one unit of an enzyme that inhibited 50% of NBT reduction and expressed as unit per milligram protein. CAT activity in the aortic samples was tested on the basis of a previous method (Aebi, 1984). The samples were mixed into H_2_O_2_, and the disappearance of H_2_O_2_ was measured spectrophotometrically at 240 nm. GSH level in the aortic tissue lysates was measured as described previously (Ellman, 1959). The samples were mixed with 5,5-dithio-bis-[2-nitrobenzoic acid] for 15 min. Subsequently, the absorbance values of the samples were measured at 415 nm using a microplate reader.

### Histological Analysis

Thoracic aortae were fixed in 10% formalin, dehydrated, and embedded in paraffin wax. Aortic sections were cut on a rotary microtome and stained with hematoxylin and eosin. The sections were photographed using an Olympus SZ61TR-TP051000 microscope (Olympus, Japan), and the images were analyzed using Life Science Olympus cellSens Standard software (Olympus, Japan). The morphometric parameters of the aorta, including intima–media thickness (IMT), intima–media area (IMA), lumen diameter (*d*), circumference wall tension (CWT), and tensile stress (TS), were measured based on previous protocols (Zainalabidin et al., 2014). The four quadrants of the aortic lumen at 0°, 90°, 180°, and 270° between the layers of the tunica media and tunica intima were measured, and the average readings were recorded as the IMT value. Lumen area (*α*) was calculated by drawing a circular line over the tunica intima layer. Lumen diameter (*d*) was calculated using the formula $d=\left(2\sqrt{\alpha }\right)/\pi$, where *π* is equivalent to 3.14. IMA was calculated using the formula: $\left[\pi {\left(d/2+IMT\right)}^{2}\right]-\left[\pi {\left(d/2\right)}^{2}\right]$. MSBP was used in the formula to calculate CWT, where CWT = MSBP × (*d*/2). Finally, TS was calculated using the formula: TS = CWT/IMT.

### Statistical Analysis

The data were analyzed using GraphPad Prism software version 7. The results are presented as mean ± standard error of the mean (SEM). The myography data were analyzed using two-way ANOVA followed by Tukey’s post hoc test. For other data, unpaired *t*-test was used to compare the means. *p* < 0.05 was considered statistically significant.

## Results

### Effect of Aqueous Extract of *Piper sarmentosum* Roxb. Leaf on Vasorelaxation

**FIGURE 1:**
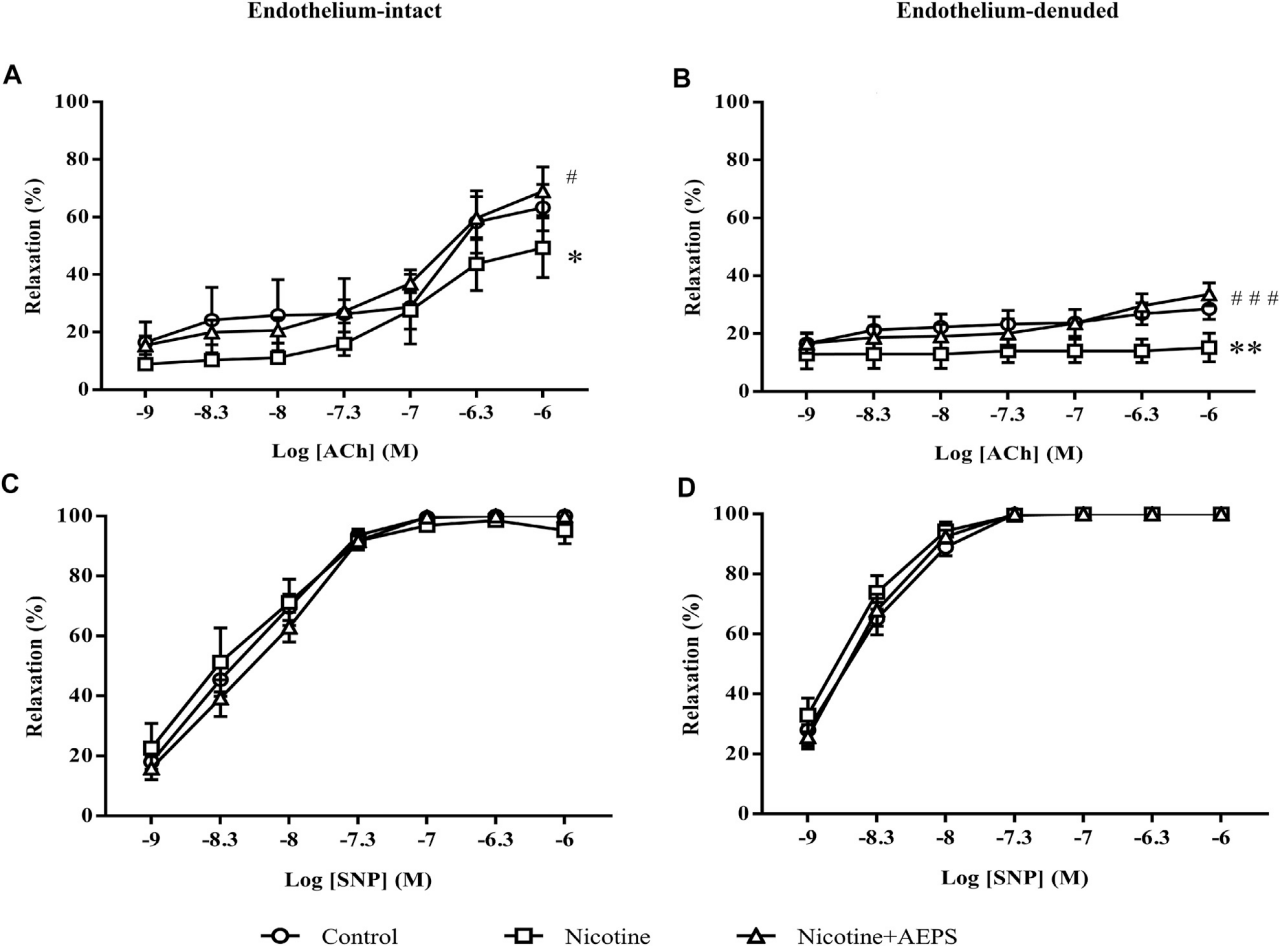
Concentration-relaxation curves to the endothelium-dependent vasodilator ACh **(A, B)** and endothelium-independent vasodilator SNP **(C, D)** in rat aortic rings from each group. The values are given as mean ± SEM, *n* = 6–8 for each group. *p < 0.05, **p < 0.01 compared with the control group; ^#^ *p* < 0.05, ^###^ *p* < 0.001 compared with the nicotine group. (See Table 1 for comparison of maximum relaxation among the groups).

The endothelium-intact aortic rings of rats treated with AEPS had higher relaxation response to ACh compared with the nicotine group (*p* < 0.05). The endothelium-intact aortic rings of nicotine-induced rats showed lower relaxation response to ACh compared with the control group (*p* < 0.05) (Figure 1A). Besides, the endothelium-denuded aortic rings of rats treated with AEPS had higher relaxation response to ACh compared with the nicotine group (*p* < 0.001). Endothelium-denuded aortic rings of nicotine-induced rats showed lower relaxation response to ACh compared with the control group (*p* < 0.01, Figure 1B). The maximum relaxation (*R*_max_) of the vessels with ACh was remarkably higher in endothelium-intact vessels than in endothelium-denuded vessels in all groups (Table 1). No remarkable difference was observed in vasorelaxation toward endothelium-independent vasodilator, SNP, in all groups (Figures 1C,D). The presence or absence of endothelium also did not affect the maximum relaxation to SNP in all groups (Table 1).

**TABLE 1:** Maximum relaxation (R_max_) of aortic rings in response to ACh and SNP.

	Endothelium-intact			Endothelium-denuded		
	Control	Nicotine	Nicotine + AEPS	Control	Nicotine	Nicotine + AEPS
ACh						
R_max_ (%)	63.30 ± 8.12**	49.27 ± 10.31*	68.94 ± 8.40**	28.60 ± 3.63	15.24 ± 4.99	33.59 ± 3.94
SNP						
R_max_ (%)	100.00 ± 0.0	100.00 ± 0.0	95.20 ± 4.41	100.00 ± 0.0	100.00 ± 0.0	100.00 ± 0.0

The values are given as mean ± SEM, *n* = 6–8 for each group. *p < 0.05, **p < 0.01 compared with the endothelium-denuded aortic rings from the similar group.

ACh, acetylcholine; SNP, sodium nitroprusside; R_max_, maximum relaxation; AEPS, aqueous extract of *Piper sarmentosum* Roxb. leaf.

### Effect of Aqueous Extract of *Piper sarmentosum* Roxb. Leaf on Vascular Nitric Oxide Level

**FIGURE 2:**
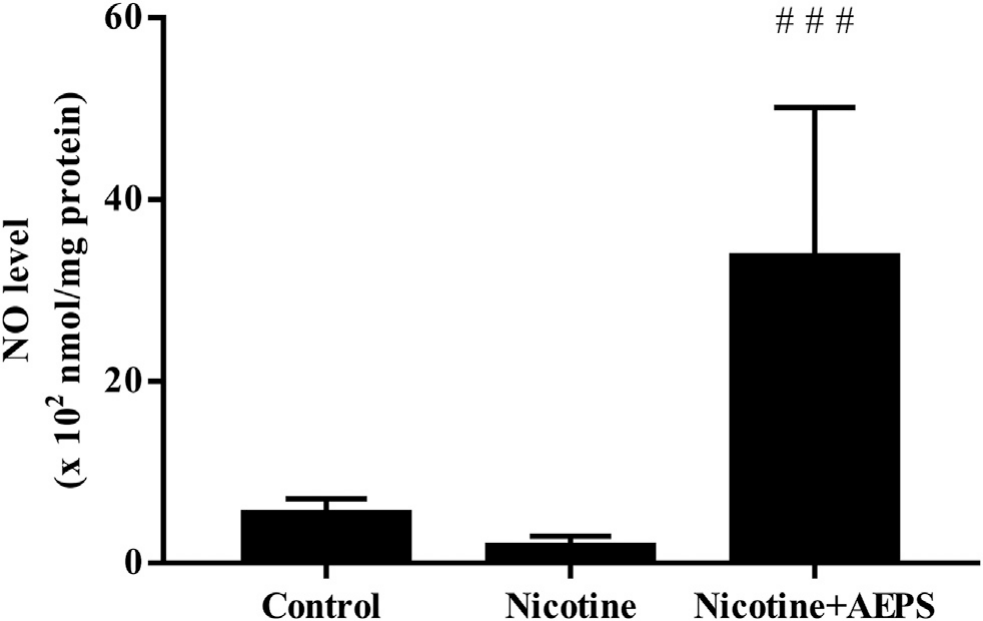
Level of nitric oxide (NO) in rat aortic tissues. The values are given as mean ± SEM, *n* = 6–8 for each group. ^###^ *p* < 0.001 compared with the nicotine group.

Rats treated with AEPS had significantly higher vascular NO level compared with nicotine-administered rats (*p* < 0.001). However, nicotine administration did not cause any remarkable change in vascular NO level compared with the control (Figure 2).

### Effect of Aqueous Extract of *Piper sarmentosum* Roxb. Leaf on Vascular Antioxidant Levels

**FIGURE 3:**
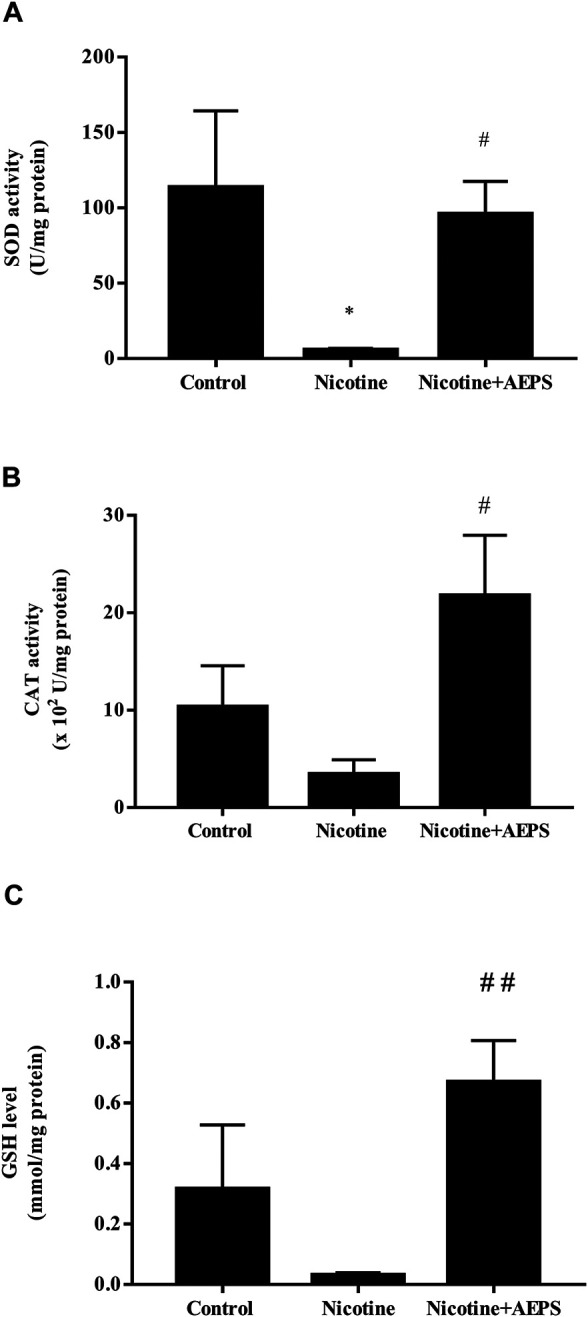
Level of antioxidants **(A)** superoxide dismutase (SOD) activity **(B)** catalase (CAT) activity and **(C)** reduced glutathione (GSH) level in rat aortic tissues from each group. The values are given as mean ± SEM, *n* = 6–8 for each group. *p < 0.05 compared with the control group, ^#^ *p* < 0.05, ^##^ *p* < 0.01 compared with the nicotine group.

Treatment with AEPS significantly improved vascular antioxidant levels as shown by the enhanced SOD activity (*p* < 0.05), CAT activity (*p* < 0.05), and GSH level (*p* < 0.05) compared with the nicotine group. Nicotine significantly reduced vascular SOD activity (*p* < 0.05) but not CAT activity and GSH levels compared with the control group (Figures 3A–C).

### Effect of Aqueous Extract of *Piper sarmentosum* Roxb. Leaf on Vascular Remodeling

**FIGURE 4:**
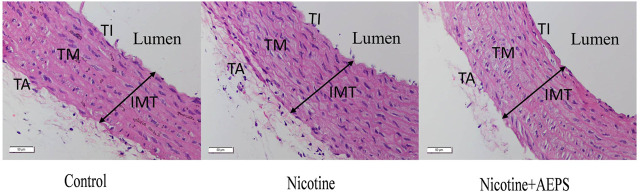
Representative images of haematoxylin and eosin-stained sections of the aortic wall from each group of rats. Control and nicotine + AEPS groups showed normal histological appearance. The nicotine group displayed disorganized tunica media layer (*n* = 6-8 per group, ×400 magnification, scale bar: 50 µm). IMT, intima-media thickness; TI, tunica intima; TM, tunica media; TA, tunica adventitia.

The aorta from the control and AEPS groups exhibited normal histology as indicated by the regular arrangement of the elastic lamella in the tunica media layer. The nicotine group displayed disorganized tunica media layer with increased interlamellar space (Figure 4). However, the morphometric analysis of the aorta, including IMT, IMA, *d*, CWT, and TS, showed no remarkable changes in all groups, even though the nicotine group showed an increasing trend in IMT (Table 2).

**TABLE 2:** Aortic morphometry measurements.

Groups	IMT (μm)	Lumen diameter (mm)	IMA (mm^2^)	CWT (× 10^4^ dyne/cm)	TS (× 10^4^ dyne/cm^2^)
Control	153.70 ± 6.12	0.78 ± 0.04	0.45 ± 0.02	4.48 ± 0.34	295.10 ± 27.75
Nicotine	170.00 ± 11.01	0.87 ± 0.03	0.59 ± 0.06	5.13 ± 0.27	310.00 ± 25.89
Nicotine + AEPS	152.80 ± 5.65	0.89 ± 0.03	0.50 ± 0.02	5.45 ± 0.26	361.50 ± 24.99

The values are given as mean ± SEM, *n* = 6–8 for each group. No significant difference was observed in any group.

IMT, intima-media thickness; IMA, intima-media area; CWT, circumferential wall tension; TS, tensile stress.

## Discussion

This study showed that AEPS attenuated nicotine-induced vascular endothelial dysfunction as AEPS improved endothelium-dependent vasorelaxation in rats administered with nicotine. Exposure to nicotine impaired endothelium-dependent vasorelaxation to ACh. Impaired endothelium-dependent vasorelaxation is a functional characteristic of vascular endothelial dysfunction (Daiber et al., 2017). The effects of nicotine and AEPS on vasorelaxation were endothelium dependent because of the remarkable reduction in vasorelaxation in response to ACh but not in response to SNP. Besides, the maximum vasorelaxation to ACh was substantially higher in endothelium-intact vessels.

ACh works by binding to M3 receptors on the endothelium, which leads to calcium release and endothelial nitric oxide synthase (eNOS) activation. eNOS converts L-arginine to NO, which diffuses from the ECs into the adjacent layer of smooth muscle to cause vasorelaxation (Sandoo et al., 2010). By contrast, SNP is a NO donor that acts directly on VSMCs to cause vasorelaxation. The response to SNP is not dependent on the production of NO by ECs (Si et al., 2017).

This study demonstrated that AEPS supplementation to nicotine-induced rats increased vascular NO level. NO is a potent vasodilator; thus, the improved vasorelaxation observed in rats treated with AEPS is most likely contributed by the enhanced NO levels. AEPS also increases NO levels in spontaneously hypertensive rats and L-N^G^-nitro arginine methyl ester-induced hypertensive rats (Zainudin et al., 2015; Alwi et al., 2018). AEPS stimulates the synthesis of NO by increasing mRNA expression, protein level, and eNOS activity in ECs (Ugusman et al., 2010). Besides, antioxidants can also protect NO from degradation by free radicals and thus maintain the bioavailability of NO (Ugusman et al., 2010). The antioxidant activity of AEPS is well established (Zainudin et al., 2015; Wang et al., 2017; Yeo et al., 2018) and may also contribute to the enhanced NO level.

Impaired vasorelaxation due to nicotine exposure has been linked to increased vascular oxidative stress (Si et al., 2017). In a previous study, nicotine reduced the relaxation of rat’s aortic rings and increased the level of oxidative stress marker, malondialdehyde (Zainalabidin et al., 2014). Oxidative stress reduces endothelial NO production by deactivating eNOS (Ugusman et al., 2010). Nicotine deactivates eNOS by reducing the availability of eNOS essential cofactor, tetrahydrobiopterin (BH_4_). BH_4_ deficiency causes eNOS to become unpaired and unable to produce NO (Forstermann and Munzel, 2006; Li et al., 2018). Apart from NO, nicotine reduces other endothelium-dependent vasodilators, such as prostaglandins and prostacyclin (Tonnessen et al., 2000). The reduction of NO and other endothelium-dependent vasodilator results in impaired vasorelaxation. However, we did not measure the level of other endothelium-dependent vasodilators in this study.

Nonetheless, in this study, nicotine did not cause a remarkable reduction in NO level. Even though the NO level in nicotine-treated rats was not substantially reduced, the nicotine group had a NO reduction trend. We used the whole aorta for the measurement of NO level without specifically isolating the endothelial layer. This factor might contribute to the non-remarkability of the results. Nevertheless, previous studies related to the effects of nicotine on NO level have shown various inconsistent results. A study showed that nicotine does not cause any change in NO (Moon et al., 2014). Another study showed that nicotine reduces NO level by deactivating eNOS and stimulating the generation of superoxide anions that degrade NO (Kathuria et al., 2013). By contrast, a previous study showed that nicotine increases NO levels by increasing intracellular calcium, which in turn activates eNOS to produce NO (Ijomone et al., 2014). These inconsistent results may be contributed by the difference in the availability of eNOS cofactors, such as BH_4_ and NADPH, which are important for eNOS activity (Ugusman et al., 2010), as well as the oxidative degradation of NO by superoxide anion (Tonnessen et al., 2000).

In view of the relationship of nicotine with oxidative stress and NO level, which contributes to vascular endothelial dysfunction, SOD activity, CAT activity, and GSH levels in the aortic tissues were measured. The results showed that supplementation with AEPS enhanced SOD and CAT activities, as well as GSH level, in the aorta of nicotine-induced rats. This result suggests a protective effect of AEPS against oxidative stress induced by nicotine. The results align with previous findings that showed that AEPS enhances the mRNA expression of SOD, CAT, and glutathione peroxidase (GPX) in ECs exposed to H_2_O_2_ (Ugusman et al., 2011).

The results also demonstrated that nicotine decreased SOD activity, but no considerable differences in CAT activity and GSH level were found. Nicotine stimulates the production of superoxide anion by increasing the expression of NADPH oxidase 4 (NOX4) (Hua et al., 2010). NOX4 is the major source of superoxide anion in blood vessels (Ugusman et al., 2011). SOD is an antioxidant enzyme that acts as the first defense to neutralize superoxide anions into H_2_O_2_ (Zainalabidin et al., 2016). The excessive usage of SOD to neutralize the superoxide anions produced in response to nicotine has led to reduced SOD activity (Moon et al., 2014; Zainalabidin et al., 2014). Additionally, CAT transforms H_2_O_2_ to oxygen and water, whereas GSH is needed for GPX to convert H_2_O_2_ into water (Holben and Smith, 1999). In the present study, nicotine did not reduce the levels of CAT and GSH. This result is probably related to the action of SOD as the first antioxidant enzyme that neutralizes excessive free radicals before the actions of CAT and GSH start (Zainalabidin et al., 2016).

Collectively, this study showed that AEPS attenuates nicotine-induced vascular endothelial dysfunction by improving vasorelaxation and increasing vascular NO and antioxidant levels. The antioxidant activity of AEPS is often associated with its flavonoid content (Lee et al., 2014). Based on the LCMS analysis, the AEPS used in this study contains flavonoids, such as quercetin, naringenin, and vitexin (Sundar et al., 2019). Quercetin and naringenin are potent antioxidants that stimulate endothelial NO production (Yamamoto and Oue, 2006; Qin et al., 2016). Quercetin improves endothelium-dependent vasorelaxation by stimulating eNOS activity and increasing NO bioavailability in endothelial cells (Shen et al., 2012). Another flavonoid found in AEPS, naringenin, restores ACh-induced vasorelaxation in the aorta of diabetic rats. Naringenin reduces diabetic vascular endothelial dysfunction by downregulating oxidative stress and inflammation (Ren et al., 2016). In addition, vitexin improves aortic relaxation to ACh in chronic myocardial ischemia/reperfusion injury rat model (Che et al., 2016). Vitexin activates eNOS via the PI3K/Akt signaling pathway and can therefore regulate NO level (Cui et al., 2019). The present study used crude AEPS and not its purified active compound; thus, we were unable to pinpoint the specific compound of the extract that mediated the positive effects. However, the protective effects of AEPS on vascular endothelial dysfunction are probably mediated by the abovementioned potential flavonoids.

Apart from impaired vasorelaxation, abnormal vascular remodeling is one of the markers of chronic vascular dysfunction (Zainalabidin et al., 2014). AEPS treatment maintained the normal histological features of the aorta, whereas nicotine disorganized the tunica media layer and increased the interlamellar space. However, the morphometric analysis of the aorta showed no remarkable changes in all groups, even though IMT tend to increase in the nicotine group. The effect of nicotine on vascular remodeling is closely related to its dose and duration (Li et al., 2017). The i.p. injection of 0.6 mg/kg/day nicotine for 28 days causes an increment in IMT and CWT and a narrowing of the aortic lumen in rats (Zainalabidin et al., 2014). Nicotine infusion at 20 mg/kg/day for 40 days results in elastin fragmentation and increased stiffness in mouse aorta (Wagenhauser et al., 2018). The dose and duration of nicotine administration in this study were 0.8 mg/kg/day and 21 days, respectively. The duration of nicotine administration in this study is probably not long enough to cause remarkable structural changes in the aortic wall. Besides, we did not incorporate a special stain, such as the Verhoeff–van Gieson stain, to delineate the elastic fibers in the aortic wall for a better morphological assessment. This factor might also affect our morphometric analysis of the aorta.

Another limitation of this study is the absence of a treatment control group. This study reported the findings from three experimental groups, namely, control, nicotine, and nicotine + AEPS groups. A treatment control group that consists of AEPS alone should be included to affirm the safety and exclude any potential adverse effects of AEPS on healthy vasculature. Nonetheless, our previous studies that incorporated a treatment control group with AEPS alone showed no adverse effect on healthy vasculature in *in vitro* and *in vivo* levels. For instance, AEPS up to 300 μg/ml concentration did not reduce endothelial cell viability (Sundar et al., 2019). In rats, treatment with 500 mg/kg/day AEPS for 28 days did not cause any remarkable change in blood pressure (Alwi et al., 2018; Ugusman et al., 2020; Azmi et al., 2021). Besides, rat aorta displayed normal histology under light and electron microscopic examinations following AEPS treatment (Thent et al., 2012a; Thent et al., 2012b). Overall, previous studies showed that AEPS has no adverse effects on healthy vasculature.

## Conclusion

*P. sarmentosum* attenuates nicotine-induced vascular endothelial dysfunction by enhancing vasorelaxation, vascular NO, and antioxidant levels. Thus, *P. sarmentosum* may be beneficial to prevent the vascular endothelial dysfunction caused by nicotine exposure. However, the molecular mechanism underlying the protective effect of *P. sarmentosum* on nicotine-induced vascular endothelial dysfunction needs further investigation. Furthermore, this study is an *in vivo* animal study that investigated some fundamental effects of AEPS on nicotine-induced vascular endothelial dysfunction. Further studies are required to identify and isolate the active compounds in AEPS responsible for the positive effects, as well as to explore their mechanisms of action. In addition, clinical trials that incorporate sufficient sample size and thorough methodology are needed to confirm our conclusions on the efficacy and safety of AEPS as a supplement for nicotine-induced vascular endothelial dysfunction in humans.

## Data Availability

The original contributions presented in the study are included in the article/Supplementary Material, further inquiries can be directed to the corresponding author.

## Glossary

ACh: acetylcholine
AEPS: aqueous extract of *Piper sarmentosum* Roxb. leaf
BH_4_: tetrahydrobiopterin
BW: body weight
CAT: catalase
CVD: cardiovascular diseases
CWT: circumference wall tension
EC: endothelial cell
EDTA: ethylenediaminetetraacetic acid
eNOS: endothelial nitric oxide synthase
GPX: glutathione peroxidase
GSH: reduced glutathione
H&E: hematoxylin and eosin
H_2_O_2_: hydrogen peroxide
i.p.: intraperitoneally
IMA: intima-media area
IMT: intima-media thickness
LCMS: liquid chromatography mass spectrometry
MMP: matrix metalloproteinase
mRNA: messenger ribonucleic acid
MSBP: mean systolic blood pressure
NAChR: nicotinic acetylcholine receptor
NADPH: nicotinamide adenine dinucleotide phosphate
NBT: nitro blue tetrazolium
NO: nitric oxide
NOX4: NADPH oxidase 4
p.o.: oral gavage
PBS: phosphate-buffered saline
R_max_: maximum relaxation
SEM: standard error of the mean
SNP: sodium nitroprusside
SOD: superoxide dismutase
TS: tensile stress
UKM: Universiti Kebangsaan Malaysia
VSMC: vascular smooth muscle cell
w/v: weight/volume
