# Supplementary material for: Piper sarmentosum Roxb. Attenuates Vascular Endothelial Dysfunction in Nicotine-Induced Rats
Source: Front Pharmacol. 2021 Jun 14;12:667102. doi: 10.3389/fphar.2021.667102 (PMC8236855; doi:10.3389/fphar.2021.667102)
Supplement: Supplementary file 1 [file Table1.pdf]

Supplementary Table 1. Maximum relaxation of aortic rings in response to ACh and SNP.

	Endothelium-intact					Endothelium-denuded				
	Control	Nicotine	Nicotine + AEPS 125 mg/kg BW	Nicotine + AEPS 250 mg/kg BW	Nicotine+ AEPS 500 mg/kg BW	Control	Nicotine	Nicotine + AEPS 125 mg/kg BW	Nicotine + AEPS 250 mg/kg BW	Nicotine + AEPS 500 mg/kg BW
ACh										
R_{max} (%)	57.25 ± 7.62*	38.05 ± 8.65*	52.52 ± 28.23	64.05 ± 9.66*	46.23 ± 6.05	25.83 ± 1.16	10.98 ± 3.67	49.74 ± 17.90	28.46 ± 5.96	25.28 ± 8.78
SNP										
R_{max} (%)	100.00 ± 00	90.39 ± 8.62	95.20 ± 4.41	99.31 ± 0.35	100.00 ± 00	100.00 ± 00	100.00 ± 00	100.00 ± 00	100.00 ± 00	100.00 ± 00

Values are given as mean ± SEM, n=3 for each group. **P* <0.05 compared to endothelium-denuded aortic rings from similar group. ACh, acetylcholine; SNP, sodium nitroprusside; R_{max}, maximum relaxation; AEPS, aqueous extract of *Piper sarmentosum.*
