# Supplementary material for: Piper sarmentosum Roxb. Attenuates Vascular Endothelial Dysfunction in Nicotine-Induced Rats
Source: Front Pharmacol. 2021 Jun 14;12:667102. doi: 10.3389/fphar.2021.667102 (PMC8236855; doi:10.3389/fphar.2021.667102)
Supplement: Supplementary file 2 [file Image1.pdf]

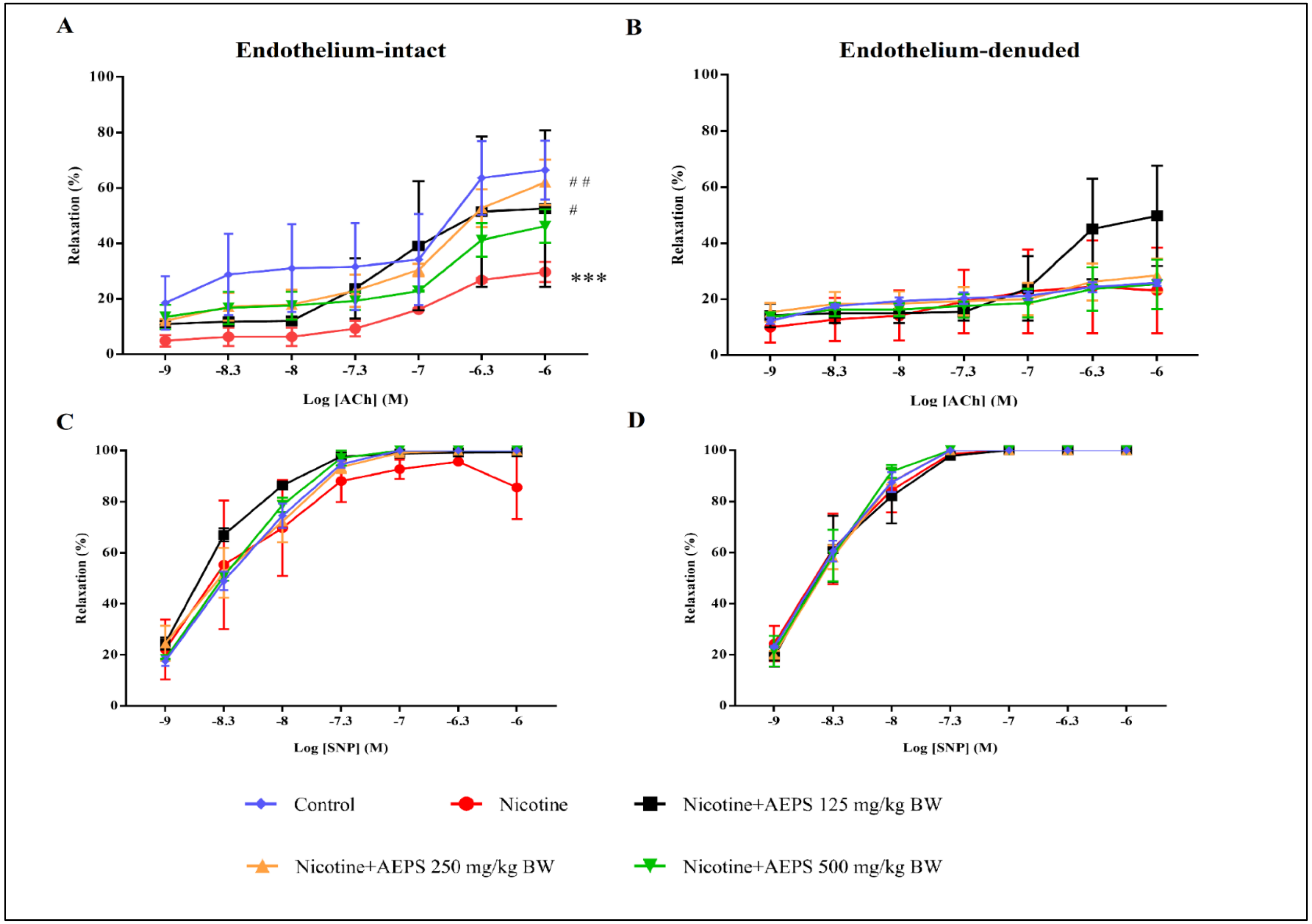

Supplementary Figure 1. Concentration-relaxation curves to the endothelium-dependent vasodilator ACh (A, B) and endothelium-independent vasodilator SNP (C, D) in rat aortic rings from each group. Values are given as mean ± SEM, n=3 for each group. ****P* <0.001 compared to control group; $^{\#}P$ <0.05, $^{\#\#}P$ <0.01 compared to nicotine group. (See Supplementary Table 1 for comparison of maximum relaxation among groups).
